# Remote Control of Gene Function by Local Translation

**DOI:** 10.1016/j.cell.2014.03.005

**Published:** 2014-03-27

**Authors:** Hosung Jung, Christos G. Gkogkas, Nahum Sonenberg, Christine E. Holt

**Affiliations:** 1 Department of Anatomy, Brain Research Institute, and Brain Korea 21 PLUS Project for Medical Science, Yonsei University College of Medicine, Seoul 120-752, South Korea; 2 Patrick Wild Centre, Centre for Integrative Physiology, Hugh Robson Building, University of Edinburgh, Edinburgh EH8 9XD, UK; 3 Department of Biochemistry and Goodman Cancer Research Centre, McGill University, Montreal, QC H3A 1A3, Canada; 4 Department of Physiology Development and Neuroscience, Anatomy Building, Downing Street, University of Cambridge, Cambridge CB2 3DY, UK

## Abstract

The subcellular position of a protein is a key determinant of its function. Mounting evidence indicates that RNA localization, where specific mRNAs are transported subcellularly and subsequently translated in response to localized signals, is an evolutionarily conserved mechanism to control protein localization. On-site synthesis confers novel signaling properties to a protein and helps to maintain local proteome homeostasis. Local translation plays particularly important roles in distal neuronal compartments, and dysregulated RNA localization and translation cause defects in neuronal wiring and survival. Here, we discuss key findings in this area and possible implications of this adaptable and swift mechanism for spatial control of gene function.

## Main Text

### Introduction

Many cellular proteins become localized to specific subcellular locations. Spatial localization enables functional compartmentalization and is important for many aspects of cell signaling and behavior. The most common mechanism for protein localization involves direct targeting of the protein itself via specific sequences such as the nuclear or mitochondrial localization sequences (Imai and Nakai, 2010). However, a large-scale in situ hybridization study in *Drosophila* embryogenesis revealed, surprisingly, that 71% of mRNAs of the genes examined (20% of total genes) localize to distinct subcellular compartments where, in many cases, they colocalize with the proteins they encode (Lécuyer et al., 2007). This remarkable finding hints at the prevalence of an alternative mechanism for protein localization: subcellular targeting of the mRNA encoding a protein and its subsequent on-site translation. This RNA-based mechanism, the focus of the current review, involves the coordination of multiple complex processes, including mRNA transport, targeting, and translation, and enables remarkably precise stimulus-driven control over protein position, abundance, and, to some extent, function.

**Figure 1:**
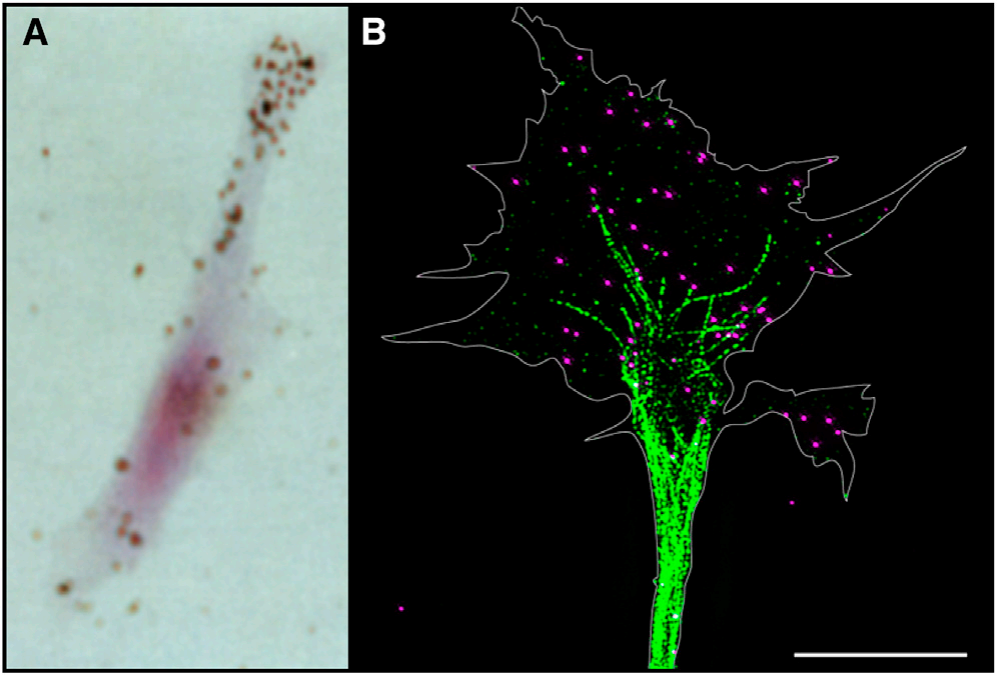
Subcellular RNA Localization in Diverse Cell Types (A) *β-actin* mRNA localizes to the periphery of migrating fibroblasts (Lawrence and Singer, 1986). (B) *β-actin* mRNA (magenta, in situ hybridization) localizes to the axonal growth cone of a retinal ganglion cell neuron from a cultured *Xenopus laevis* eye primordium and partially colocalizes with the dynamic microtubule cytoskeleton (green, anti-tyrosinated tubulin). Superresolution image acquired using a DeltaVision OMX 3D-Structured-Illumination Microscope (Applied Precision), with a 100× 1.4 NA oil objective. Extended focus image from deconvolved Z stack images acquired at 0.125 μm step size B. Lu and C.E.H., unpublished). Scale bar, 5 μm.

Subcellular RNA localization is highly prevalent in eukaryotes, ranging from yeast (Gonsalvez et al., 2005) to highly specialized cells such as neurons (Bramham and Wells, 2007; Jung et al., 2012; Sutton and Schuman, 2006) and oligodendrocytes (Hoek et al., 1998), and it is also found in bacteria (Keiler, 2011). Neurons serve as an excellent model to understand RNA localization as they are highly polarized: the distal tip of the neuronal axon is remote from its cell body, sometimes a meter away, and therefore can be easily isolated (Campenot and Eng, 2000; Taylor et al., 2009; Zivraj et al., 2010). Comparative subcellular transcriptome analyses in neuronal processes have revealed that distinct sets of mRNAs are targeted to different compartments (Andreassi et al., 2010; Cajigas et al., 2012; Gumy et al., 2011; Minis et al., 2014; Taylor et al., 2009; Zivraj et al., 2010). This novel layer of intracellular patterning, originally thought to be exclusive to highly specialized cells where it was first discovered (Lasko, 2012), may occur widely in many cell types, as suggested by the localization of subsets of mRNAs to cell protrusions in migrating fibroblasts (Lawrence and Singer, 1986; Mili et al., 2008) (Figure 1).

RNA localization may be an evolutionarily conserved mechanism that decentralizes genomic information and delegates its control to subcellular compartments (Holt and Schuman, 2013). The genetic information encoded in the nucleus provides the supply of mRNAs by transcription from which specific sets of mRNAs are chosen for subcellular localization. Chosen mRNAs are targeted to multiple locations while their translation is repressed during their transit (Erickson and Lykke-Andersen, 2011; Krichevsky and Kosik, 2001). The composition of transported mRNAs is regulated by both cell-intrinsic (Gumy et al., 2011; Taylor et al., 2009; Zivraj et al., 2010) and -extrinsic signals (Dictenberg et al., 2008; Mingle et al., 2005; Willis et al., 2007). Thus, mRNAs are much more than simple “messengers” that deliver the genetic information from DNA to the protein synthetic apparatus, inasmuch as subcellularly targeted collections of mRNAs can function as a genomic outpost. There, functionally related mRNAs can be synchronously translated according to biological needs, providing an efficient means for coordinate control of gene expression (Keene and Tenenbaum, 2002), comparable to the efficient bacterial operon system (Jacob et al., 1960). Moreover, it is becoming increasingly clear that dysfunctional RNA localization and translation represent one of most common molecular pathologies of neurodevelopmental and neurodegenerative diseases (Kelleher and Bear, 2008; Jung et al., 2012; Liu-Yesucevitz et al., 2011; Ramaswami et al., 2013; Wang et al., 2007).

In this review, we present localized translation as a distinct mode of gene expression control that positions gene function with extreme spatiotemporal precision, efficiency, and flexibility. We assess current knowledge of how distinct subsets of mRNAs might be targeted to subcellular locations where they await a signal to proceed synthesizing proteins and relate how these RNA-based mechanisms might be linked to general biological themes of Location Matters and Decoding the Brain, which are covered elsewhere in this special reviews issue.

### Biological Function of Local Translation

Synthesizing a protein where and when it is needed provides several advantages over transporting a pre-existing protein from one place in the cell to another. Local synthesis confers ultimate precision in protein localization, as a protein is present only where it is needed and not anywhere else. On-site synthesis instantly satisfies the biological demand for a protein without any delay in its transport. Additionally, removing the reliance on a protein-encoded transport signal means that the same protein can be targeted to diverse subcellular compartments without risking changing its structure or compromising its function. In this latter case, the localization information can be encoded in the mRNA untranslated region (UTR) rather than in the protein-coding region. Finally, many copies of proteins can, in theory, be made from one mRNA molecule by multiple rounds of translation conferring an economical advantage.

Newly made proteins can play at least two roles. First, they harbor unique information distinct from pre-existing ones, and thus can be used to deliver an additional layer of signaling information (Holt and Bullock, 2009). An alternative, but not mutually exclusive role of newly made proteins is to replenish damaged, degraded, or inactivated proteins to maintain local proteome homeostasis.

#### Properties Unique to Newly Made Proteins Provide Signaling Information

*Location*. The location of the birth of a protein encodes important signaling information. The growth cone, the tip of a growing axon, is thought to be nature’s most sensitive sensor of chemical gradients as it can detect a concentration difference as little as 0.1% (Rosoff et al., 2004). When challenged with a gradient of an attractive guidance cue, netrin-1 or brain-derived neurotrophic factor (BDNF), asymmetric translation of *β-actin* mRNA occurs within the growth cone on the side nearest the source of the gradient (Leung et al., 2006). This precise spatial regulation of mRNA translation (growth cones are around 5 μm across) precedes and is required for the growth cone’s ability to turn toward the source of the guidance cue (Leung et al., 2006; Yao et al., 2006). In neuronal dendrites, on an even smaller scale, synaptic activation induces transcript-specific translation only at the stimulated synapses (Wang et al., 2009), allowing context-dependent, spatially restricted changes in synaptic structure and function.

There is evidence that transcription factors—such as CREB (cyclic AMP-responsive element-binding protein) (Cox et al., 2008), STAT3 (signal transducer and activator of transcription 3) (Ben-Yaakov et al., 2012), and SMADs (homologs of *C. elegans s*mall body size and *Drosophila m*others *a*gainst *d*ecapentaplegic) (Ji and Jaffrey, 2012)—synthesized in distal axons interact with the local signaling milieu immediately after their synthesis, and carry this unique birth-place information to the nucleus where it modulates their gene regulatory function.

Another intriguing case, although it remains to be corroborated, is the recent report of nuclear translation. Peptides encoded in the intron are generated by an unknown mode of translation before pre-mRNA splicing and subsequent mRNA nuclear export. The peptides are presented to the major histocompatibility complex (MHC) I pathway in cells expressing all possible splice variants during T-cell-negative selection, thus preventing autoimmune reactions (Apcher et al., 2013).

*Time*. The time of the birth of a protein can also encode signaling information. Many proteins are subject to posttranslational modifications as they execute their functions or even as they age. Newly synthesized proteins thus are distinct from their existent counterparts in several important aspects. For example, little or no posttranslational modification of a “new” β-actin molecule provides information distinct from that of “old” ones that have been posttranslationally modified by glutathionylation (Wang et al., 2001) or arginylation (Karakozova et al., 2006). A highly localized, sudden rise in nascent β-actin molecules, which are likely to have a faster rate of polymerization than pre-existing β-actins, may link the site of local protein synthesis and actin nucleation (Condeelis and Singer, 2005).

#### Local Proteome Homeostasis

In addition to generating highly localized signaling information, localized mRNA translation is used to maintain local proteome homeostasis (Alvarez et al., 2000). The mRNA encoding the activated leukocyte cell adhesion molecule (ALCAM) localizes to the neuronal axon. There, its translation is regulated by the *cis*-element residing in its 3′-UTR (Thelen et al., 2012). ALCAM mediates homophilic adhesion of axons from the same neuronal subtype and is required for the formation of axon bundles. Excess ALCAM leads to axon bundle aggregation and prevents axonal growth, whereas too little ALCAM leads to defasciculation. Intriguingly, introduction of exogenous full-length *ALCAM* mRNA does not result in overexpression of ALCAM protein in axons, whereas the *ALCAM* mRNA lacking the 3′-UTR does, indicating that a mechanism exists to maintain the right amount of ALCAM proteins on the axonal surface by local translation.

This process has parallels with the resensitization of neuronal axons to extrinsic cues. Neuronal axons navigating toward a gradient of an attractive guidance cue must maintain the ability to respond to the pre-encountered cue. The initial encounter with an extrinsic cue leads to endocytosis of the activated receptors, and local translation is required to compensate for the loss of receptors from the axonal surface and to regain the ability to respond to the same cue, a process known as adaptation (Piper et al., 2005).

Similarly, localized translation coupled to nonsense-mediated decay, a process that degrades mRNAs containing a premature termination codon (PTC) preceding an exon junction complex (EJC) and that is activated after the first round of translation, maintains the right amount of Robo3.2 receptor in neuronal axons to position commissural neuronal axons in the appropriate place (Colak et al., 2013). Neuronal axons can synthesize locally a significant amount of diverse proteins (10% synthesis per unit volume compared to the cell body cytoplasm) (Lee and Hollenbeck, 2003), suggesting that localized translation may provide a quality control mechanism that ensures the optimal amount of a protein is expressed in subcellular compartments.

### Molecular Control of Translation

As described above, positioning the relevant mRNAs at the appropriate place within a cell enables an accelerated response to signaling inputs. With mRNAs stockpiled at distinct locations, there is little time spent moving proteins through large regions of cytoplasm. Translational activation of selected mRNAs at sites within cells draws on established control mechanisms.

**Figure 2:**
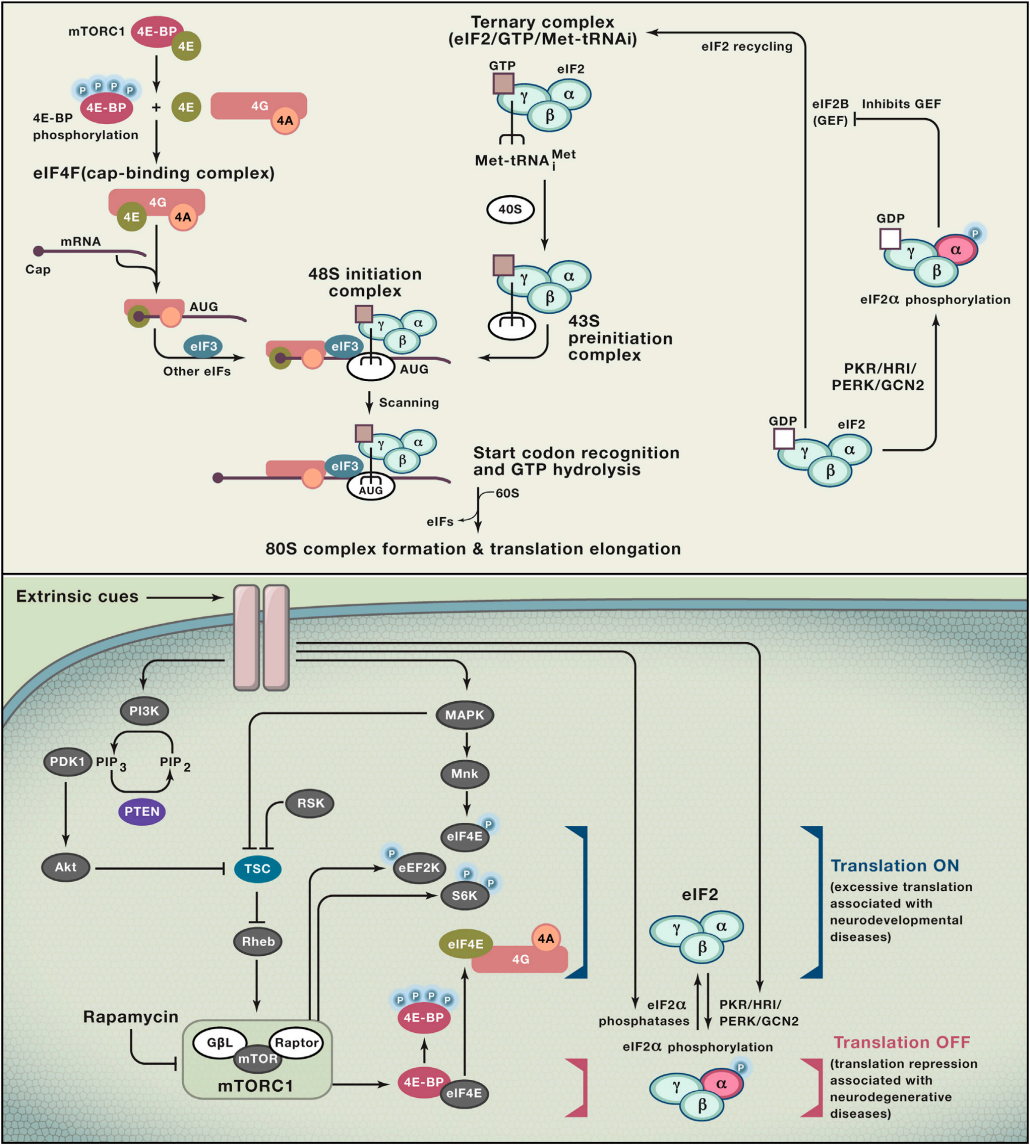
Translational Control in Mammals (A) Cap-dependent translation (top). Translation in mammals is mainly regulated at the initiation step. The cap-binding eIF4F complex, downstream of mTORC1, and the 43S preinitiation complex are the major targets of translational control. (B) Regulation of cap-dependent translation (bottom). mTORC1 links cell-intrinsic and -extrinsic signals to translation initiation by phosphorylating S6Ks and 4E-BPs. eIF2α phosphorylation represses translation. Excessively high or low translation activities are associated with neurodevelopmental and neurodegenerative diseases, respectively (see text for more details).

Translational control provides a powerful means to induce rapid changes in protein amounts and, indeed, the abundance of a protein in mammalian cells can be best predicted by the rate of mRNA translation rather than by mRNA abundance (Schwanhäusser et al., 2011). Translation is controlled via a large number of mechanisms (reviewed in Sonenberg and Hinnebusch, 2009), including changes in the amounts and activities of translation components: ribosomes, translation factors and tRNAs. The best understood regulatory step is the phosphorylation of translation factors and their regulators, particularly that of key eukaryotic translation initiation factors (eIFs). All eukaryotic nuclear-transcribed mRNAs possess a 5′-end cap structure. Two macromolecular complexes that function in cap-dependent translation initiation, the eIF4F and the 43S preinitiation complex, are the major targets of the translational regulation (Figure 2A).

#### Regulation via eIF4F, the Cap-Binding Complex

eIF4F is a heteromeric complex (Edery et al., 1983; Grifo et al., 1983) that binds the cap structure and is composed of eIF4A (RNA helicase), eIF4E (cap-binding protein) (Sonenberg et al., 1979) and eIF4G (scaffolding protein) that binds both eIF4E and eIF4A (Figure 2A). After binding to the cap, eIF4F unwinds the mRNA 5′-proximal secondary structure to facilitate the binding of the 43S preinitiation complex (see below).

*eIF4F Formation Is Regulated by the Phosphorylation Status of 4E-BPs*. Because eIF4E generally exhibits the lowest expression level of all eIFs, the cap-recognition step by eIF4E is rate limiting for translation and a major target for regulation (Gingras et al., 1999). The best characterized mechanism that controls the incorporation of eIF4E into the cap-binding complex is that exerted by members of the eIF4E-binding protein (4E-BP) family: 4E-BP1, 4E-BP2, and 4E-BP3 (Pause et al., 1994). 4E-BPs and eIF4G share a common eIF4E-binding motif, through which they compete for the binding to eIF4E (Figure 2A). Hypophosphorylated 4E-BPs bind eIF4E preventing it from associating with eIF4G to form the eIF4F complex (Gingras et al., 1999).

*mTORC1 Phosphorylates 4E-BPs*. Phosphorylation of 4E-BPs releases eIF4E to promote the formation of the eIF4F complex and is, therefore, one of the rate-limiting steps in cap-dependent translation (Gingras et al., 1999). 4E-BPs’ phosphorylation is mainly controlled by the target of rapamycin (TOR), an evolutionarily conserved serine-threonine protein kinase of the phosphatidylinositol 3-kinase (PI3K)-related kinase family (Laplante and Sabatini, 2012) (Figure 2B). TOR acts as a major sensor that integrates extracellular inputs, such as insulin, growth factors, and nutrients, and intracellular levels of oxygen, energy levels, and amino acids to effect outputs including cell growth, proliferation and autophagy (Hay and Sonenberg, 2004). *Tor* genes were first discovered in yeast following a forward genetic screen for mutations that confer resistance to the antifungal agent rapamycin (Heitman et al., 1991), and subsequently its homologs were identified in mammals (mammalian/mechanistic target of rapamycin; *mTOR*) (Brown et al., 1994). mTOR is the catalytic subunit of two distinct multiprotein complexes, mTORC1 and mTORC2.

mTORC1 directly phosphorylates 4E-BPs (Brunn et al., 1997). Upon phosphorylation by mTORC1, 4E-BPs dissociate from eIF4E allowing it to form the eIF4F complex and to stimulate translation. mTORC1 also phosphorylates S6 kinase (S6K) 1 and 2, which in turn phosphorylate regulators of translation initiation such as S6, eIF4B, and PDCD4 (programmed cell death 4) and to control their activities (Laplante and Sabatini, 2012). mTORC1 may also promote translation elongation by phosphorylating eEF2K (eukaryotic elongation factor 2 kinase) that phosphorylates eEF2 (eukaryotic elongation factor 2) (Figure 2B) (Laplante and Sabatini, 2012).

*mTORC1 Pathway*. The activity of mTORC1 is controlled by the small GTPase Rheb (Ras homology enriched in brain), which is negatively regulated by its GTPase activating proteins tuberous sclerosis complex (TSC) 1 and 2. TSCs are inactive when phosphorylated, and kinases that phosphorylate TSCs, including Akt (also known as protein kinase B), ribosomal S6 kinase (RSK) 1, and mitogen-activated protein kinase (MAPK)/extracellular signal-regulated kinase (ERK) (Laplante and Sabatini, 2012), are thus positive regulators of cap-dependent translation. Phosphatase and tensin homolog (PTEN) dephosphorylates phosphatidylinositol (3,4,5)-trisphosphate (PIP3) to form phosphatidylinositol (4,5)-bisphosphate (PIP2), and thus is a negative regulator of mTORC1 (Figure 2B) (Hay and Sonenberg, 2004). MAPK/ERK phosphorylates, in addition to TSC1/2, MAP kinase-interacting kinase (Mnk) 1 and 2. Phosphorylated Mnks then bind to eIF4G and phosphorylate eIF4E, promoting cap-dependent translation (Buxade et al., 2008) (Figure 2A).

*Localized mTORC1 Activation Leads to Localized Translation*. The activation of cell-surface receptors, such as those for neurotransmitters, hormones, neurotrophic factors, and extracellular matrix components, links extrinsic signals to localized translation. For example, the synaptic activation of TrkB, a receptor for brain-derived neurotrophic factor (BDNF), leads to phosphorylation of TSCs in the dendritic spine and thus the localized activation of cap-dependent translation (Takei et al., 2004). mTORC1-dependent local protein synthesis in the dendritic spine is required for the input-specific strengthening of synaptic activity and the establishment of the late phase of long-term potentiation (LTP), a cellular substrate of learning and memory (Tang et al., 2002). Thus, coupling of cell-surface receptors to mTORC1 transduces extrinsic cues into localized protein synthesis (Graber et al., 2013; Hoeffer and Klann, 2010).

#### Control of 43S Ribosomal Preinitiation Complex Formation

eIF2 (composed of α, β and γ subunits) is a GTPase that forms a ternary complex with guanosine-5′-triphosphate (GTP) and the initiator Met-tRNA_i_^Met^. The ternary complex, together with other eIFs, binds the 40S ribosomal subunit to form the 43S preinitiation complex (Hinnebusch and Lorsch, 2012). The cap-binding complex eIF4F recruits the 43S preinitiation complex and forms the 48S initiation complex, which traverses the 5′-UTR in the 5′ to 3′ direction until it encounters the initiation codon. There, eIF2 hydrolyzes GTP to guanosine diphosphate (GDP) and inorganic phosphate, followed by the 60S subunit recruitment resulting in the formation of the 80S ribosomal complex and the release of eIFs (Figure 2A).

*Selective Control of Translation by eIF2α*. To sustain cap-dependent translation, GTP-bound eIF2 must be replenished, and this is done by eIF2B, the guanine nucleotide exchange factor (GEF) for eIF2. Phosphorylation of the α subunit of eIF2 (eIF2α) converts it into a dominant-negative inhibitor of eIF2B and therefore decreases general translation (Mohammad-Qureshi et al., 2008). In higher eukaryotes, the phosphorylation of eIF2α is controlled by four protein kinases: the double-stranded (ds) RNA-activated protein kinase (PKR), which is activated by dsRNA; the hemin-regulated inhibitor kinase (HRI), which is activated by heme deficiency; the pancreatic eIF2α or PKR-endoplasmic reticulum (ER)-related kinase (PEK/PERK), which is activated by misfolded proteins in the ER; and the general control nonderepressible-2 (GCN2), which is activated by amino acid deprivation and UV irradiation (Figure 2B). These kinases, activated in response to virus infection, iron deficiency, protein aggregation and nutritional deprivation, thus shut down protein synthetic machinery in response to various forms of cellular stress. Paradoxically, the translation of a small subset of mRNAs, which contain upstream open reading frames (uORFs) in their 5′−UTRs, is stimulated when eIF2α is phosphorylated (Hinnebusch, 1984). Such mRNAs include *Atf4* mRNA (Costa-Mattioli et al., 2005; Harding et al., 2000), whose localized translation in dendritic spines may inhibit long-term potentiation and memory formation (Costa-Mattioli et al., 2007).

#### Cap-Independent Translation

Eukaryotic mRNAs can also be translated by cap-independent mechanisms, and one way is through an internal ribosome entry site (IRES) of an mRNA. IRESs were first identified and characterized in picornavirus RNAs, which do not possess a 5′-cap structure (Hellen and Sarnow, 2001). Although several dendritically localized mRNAs contain IRES elements, how important and extensive IRES-mediated translation in vertebrates remains to be determined (Thompson, 2012).

Repeat associated non-AUG (RAN) translation is another mode of noncanonical translation, which initiates at trinucleotide repeat tracts in the absence of an initiating AUG, or near-cognate codon (Zu et al., 2011). Recently, it was shown that the expansion of CGG repeats in the 5′-UTR of the *Fmr1* gene, which is closely associated with fragile X-associated tremor/ataxia syndrome (FXTAS), leads to the production of polyglycine or polyalanine by RAN translation (Todd et al., 2013), whose spontaneous aggregation may contribute to the FXTAS pathologies.

Our understanding of the translation mechanisms comes from studies using the cell body cytoplasm, and it is possible that localized translation might employ additional modes of translational control. New techniques to visualize, isolate and analyze the translational machinery specifically from different subcellular compartments will help to address this intriguing possibility.

### mRNA-Specific Translation by Extrinsic Cues

Which particular mRNAs are selected for translation determines how a cell will react to a signal. For example, whereas attractive guidance cues (e.g., BDNF and netrin-1) stimulate local translation of the *β-actin* mRNA in the growth cone (Leung et al., 2006; Yao et al., 2006), repulsive cues (e.g., Sema3A and Slit2b) induce local translation of mRNAs encoding proteins that promote actin disassembly, such as cofilin (Piper et al., 2006) and RhoA (Wu et al., 2005). The “differential translation model” of growth cone steering thus predicts that the identity of mRNAs selected for local translation determines the direction of turning in response to extrinsic signals (Lin and Holt, 2007). Such stimulus-driven mRNA-specific local translation spatiotemporally links signal reception to gene function, and how this mRNA-specific translation is regulated is a critical unanswered question.

#### mRNAs Are Transported and Stored in mRNP Granules

**Figure 3:**
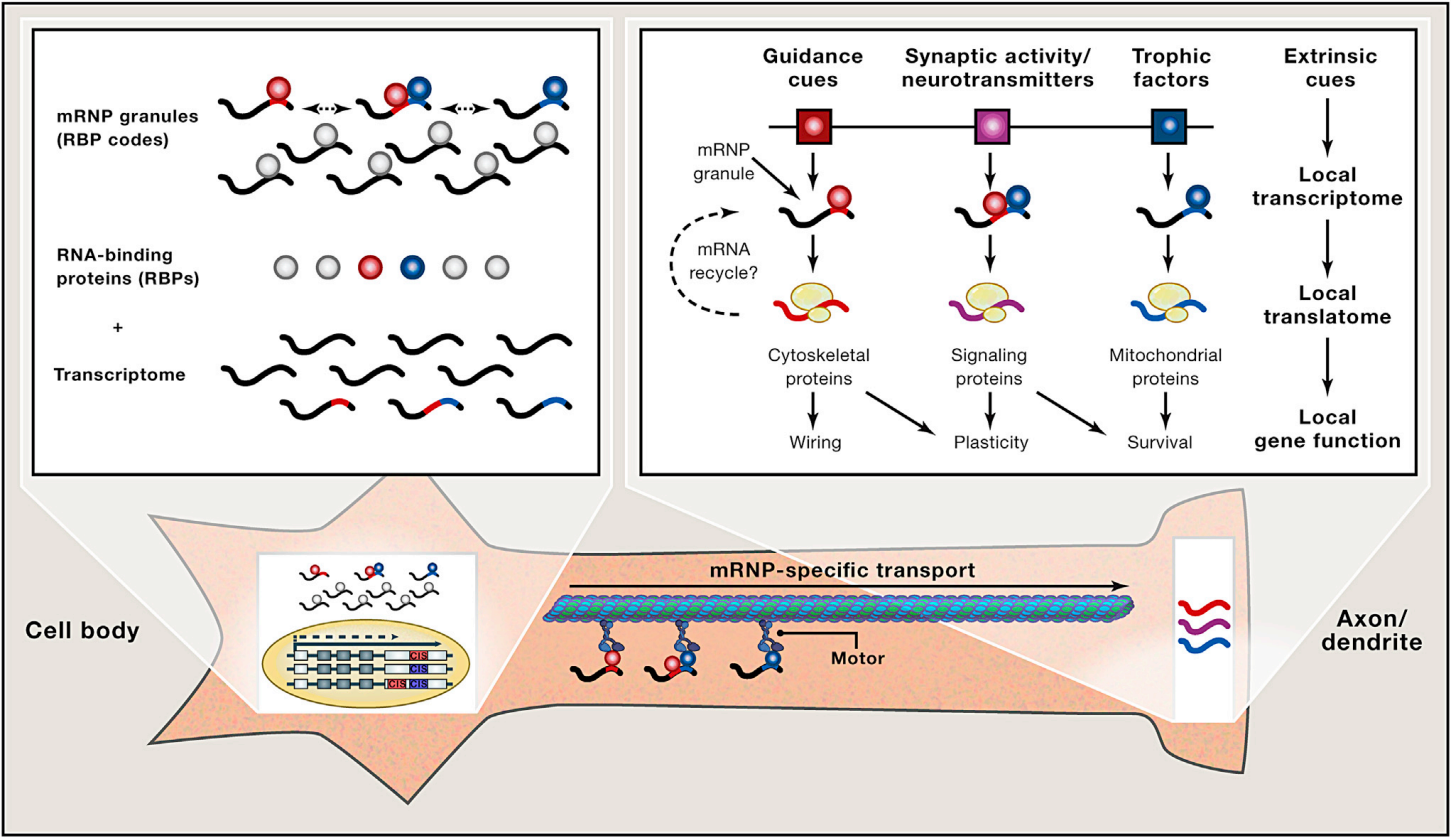
Localized Gene Expression by Translational Control of Localized mRNAs mRNAs and RNA-binding proteins form heterogeneous mRNP granules, which are transported to subcellular compartments in a translationally repressed state. Of locally stored mRNAs (local transcriptome), a selected pool of mRNAs is translated (local translatome) depending on the signaling input.

To understand how mRNA-specific translation occurs, it is important to consider how different mRNAs are transported to specific subcellular compartments since the pool of resident mRNAs will limit selection. Directed transport of an mRNA requires engagement of cytoskeletal motors (Figure 3). Cellular mRNAs are associated with RNA-binding proteins (RBPs), which directly or indirectly bind to motor proteins in membraneless, high molecular weight messenger ribonucleoprotein (mRNP) granules, which contain mRNAs, proteins and regulatory RNAs. The composition of an mRNP determines the subcellular localization of its mRNA components (Erickson and Lykke-Andersen, 2011). One of the best examples was documented in budding yeast, in which Puf3, a Pumilio RBP family member, specifically binds to mRNAs encoding mitochondrial proteins (Gerber et al., 2004), and transports them to the mitochondria, ensuring that the proteins are made where they will be used (García-Rodríguez et al., 2007). Similarly, other Pumilio family members bind to mRNAs encoding distinct, functionally related proteins, such as components of the spindle and nucleolar bodies (Gerber et al., 2004).

It has become clear that many mRNAs are earmarked for specific compartments from their birth in the nucleus, as all major nuclear events—transcription, pre-mRNA splicing and nuclear export—deposit specific components to an mRNP, which affect its localization (Erickson and Lykke-Andersen, 2011). In budding yeast, restricted localization of *Ash1* mRNAs to the bud tip of the daughter cell ensures its expression there, and the disruption of *Ash1* mRNA interaction with She2p, an RBP that shuttles between the nucleus and cytoplasm, specifically in the nucleus results in diffuse localization of *Ash1* mRNA throughout the whole bud and impaired sorting of Ash1 protein (Shen et al., 2009). In addition, the nuclear pre-mRNA cap-binding complex protein CBP80 is bound to dendritically localized mRNPs, indicating that dendritically targeted mRNPs are assembled in the nucleus and transported in a translationally repressed form (di Penta et al., 2009).

#### mRNP Is a Reversibly Self-Assembling Macromolecular Complex

Knowledge of the composition of mRNP granules could be relevant to the understanding of mRNA transport. Processing bodies (P-bodies) and stress granules are the best understood classes of mRNP granules. P-bodies contain multiple mRNAs, mRNA decapping proteins such as Dcp 1 and 2, and translational repressors such as Lsm proteins, which are thought to play a role in storage and/or degradation of mRNAs, although their exact functions remain unclear. Stress granules, by contrast, contain the small ribosomal subunit, translation initiation factors, the poly(A)-binding protein (PABP), and the translational repressors TIA-1 and TIAR, and are thought to play a role in storing mRNAs whose translation is stalled at the initiation step. The neuronal RNA granule is an mRNP that transports mRNAs in neurons and contain distinct RBPs with little overlap with stress granules or P-bodies (Liu-Yesucevitz et al., 2011). Recent proteomic studies comparing the composition of two neuronal RNPs, one containing Staufen and the other Barentsz, have shown that neuronal RNA granules are highly heterogeneous and potentially bind to specific mRNAs (Fritzsche et al., 2013). Although these three types of mRNP granules—stress granules, P-bodies and neuronal RNA granules—can be distinguished by specific markers, their function appears to be similar (Erickson and Lykke-Andersen, 2011). Indeed, submicroscopic mRNPs, transport mRNPs, stress granules, P-bodies, neuronal RNA granules, and translating ribosomes may simply represent different phases of a dynamic mRNP life cycle.

Intriguingly, many RBPs, such as the P-body component Lsm4 and the stress granule component TIA-1, contain low-complexity, prion-like domains, which have a tendency to spontaneously assemble into a high molecular weight complex through parallel alignment of these domains. This self-assembling property may be a universal feature of mRNP granules, as the domains alone can form an amyloid-like hydrogel in a cell-free system (Kato et al., 2012). The assembly and disassembly of mRNPs may be regulated, as exemplified by hydrogel formation and reversal by temperature changes (Kato et al., 2012) and phosphorylation (Han et al., 2012), providing a novel conceptual basis for reversible mRNP formation. mRNA selectivity of mRNPs appears to be a property of RBPs, as hydrogel formed from purified low-complexity sequence domains of FUS, an RBP associated with the pathogenesis of amyotrophic lateral sclerosis (ALS), preferentially binds to known mRNA components of mRNP granules in neurons— mRNAs with long 3′-UTRs (Han et al., 2012).

Different mRNAs are transported and stored together. Live imaging of fluorescently labeled mRNAs injected into hippocampal neurons in culture revealed that mRNAs whose translation mediates activity-dependent synaptic plasticity, *CaMKII α*, *neurogranin*, and *Arc* mRNAs, colocalized to the same mRNP granules containing the RBP hnRNPA2 in neuronal dendrites (Gao et al., 2008). While it requires more corroboration (see Perspectives and Future Directions), these results suggest that functionally related mRNAs might be multiplexed for targeting and storage.

#### Transported mRNAs Contain cis-Acting Elements and Are Translationally Inactive

The regulatory elements that control mRNA localization must be encoded in the mRNA itself, and indeed, the 3′-UTR is where many localization elements lie. The *cis*-elements can be primary nucleotide sequences, such as those in the 3′-UTRs of *β-actin*, *RhoA*, *EphA2*, *CoxIV*, and *Impa-1* mRNAs (Andreassi et al., 2010; Aschrafi et al., 2010; Bassell et al., 1998; Brittis et al., 2002; Zhang et al., 2001), or secondary structures such as the hairpins found in the 3′-UTR of *bicoid* mRNA in *Drosophila* oocytes (Macdonald and Struhl, 1988) and *Ash1* mRNA in the budding yeast (Chartrand et al., 1999). *Cis*-elements, however, can also be localized to the 5′-UTRs as in *kor* mRNA (Tsai et al., 2007); to the protein-coding sequence as in the target mRNAs of the RBP fragile X mental retardation protein (FMRP) (which is encoded by *Fmr1* gene) (Ascano et al., 2012; Darnell et al., 2011) and *Robo3* mRNA (Kuwako et al., 2010); or to introns as in some dendritically targeted mRNAs (Buckley et al., 2011). mRNAs may be localized during translation as the signal peptide in nascent proteins can target entire translating mRNP complexes (Eliyahu et al., 2010; Kilchert and Spang, 2011).

Targeting of intron-retaining mRNAs to dendrites demonstrate that they do not undergo nonsense-mediated decay (Behm-Ansmant et al., 2007). This, together with the findings that the nuclear capping proteins CBP20/80 and EJC components localize to and are required for the proper localization of cytosolic mRNPs, suggests that mRNA targeting begins in the nucleus and that mRNAs are transported to their destination without being translated (Figure 3). The presence of in-frame stop codons in the retained introns of dendritically targeted mRNAs prompted the suggestion of cytoplasmic splicing (Buckley et al., 2011), a notion (König et al., 2007) that is not universally supported. While this idea has been strongly challenged (Friend et al., 2008; Pessa et al., 2008; Singh and Padgett, 2009), splicing activity was also reported in enucleated platelets (Denis et al., 2005) and dendrites (Glanzer et al., 2005). Local splicing in cytoplasmic subcellular compartments to generate transcript variants and regulate gene expression was suggested by Eberwine, and coined the “RNA sentinel hypothesis” (Buckley et al., 2014), which is a tantalizing idea that merits further studies.

#### Combinatorial Modularity of mRNPs Generates Diversity and Specificity

How are thousands of distinct mRNAs targeted to different subcellular locations? Most localization elements bind specific *trans*-acting factors such as RBPs. Well-studied examples of RBPs and their cognate-binding sites include cytoplasmic polyadenylation element-binding proteins (CPEBs) that are translational repressors, recognizing a specific sequence element CPE (Richter, 1999), and Staufen (Ferrandon et al., 1997) and FMRP (Darnell et al., 2001) that bind to stem-loop and G-quartet structures present in their target mRNAs, respectively. Distinct microRNAs are localized to specific subcellular compartments (Han et al., 2011; Kaplan et al., 2013; Sasaki et al., 2014), suggesting that they regulate the composition of locally translatable mRNAs by degrading or silencing their target mRNAs. Localization elements exhibit modularity, as they are functional even when ectopically fused to a reporter mRNA (Eom et al., 2003). Many mRNAs contain multiple *cis*-elements exhibiting both redundancy and diversity. For example, *bicoid* mRNA contains five redundant stem-loop structures in the 3′-UTR, which have additive effects on the anterior localization of *bicoid* mRNA in *Drosophila* oocytes (Macdonald et al., 1993). *CamKII*α mRNA, which is dendritically localized in mature neurons, contains several distinct *cis*-elements including a primary sequence CPE (Huang et al., 2003) and a secondary structure G-quartet (Subramanian et al., 2011). This combinatorial modularity of *cis*-elements and their binding partners may generate RBP codes, not unlike the transcriptional code specifying the fate of the cell bearing them (Briscoe et al., 2000). The RBP code dictates where an mRNP will be localized, as dynamic interaction of RBPs with different cytoskeletal motor proteins contributes to the localization of mRNPs (Liu-Yesucevitz et al., 2011) (Figure 3). Additional diversity may come from tuning RBPs toward different mRNAs, as the ELAV/HuB RBP binds to different sets of mRNAs after a progenitor cell differentiates into a neuron (Tenenbaum et al., 2000).

#### Stimulus-Specific mRNA Translation

The above features of mRNPs provide a conceptual basis for the notion of subcellular RNA operons by generating a manageable diversity of localized mRNAs from which those encoding functionally-related proteins are chosen for translation on demand. Indeed, synchronous translation of related mRNAs occurs during the immune response (Levine et al., 1993), upon cellular stress (Gorospe, 2003), in circadian rhythm (Garbarino-Pico et al., 2007) and during synaptic communication (Zalfa et al., 2003). The target-derived cue engrailed-1, when added to retinal axons that were severed from their cell bodies, stimulates translation of some mRNAs such as *lamin B2,* but represses that of others such as *hsp70* (Yoon et al., 2012). As previously mentioned, attractive and repulsive guidance cues induce the translation of mRNAs encoding proteins promoting actin assembly and disassembly, respectively (Leung et al., 2006; Piper et al., 2006; Wu et al., 2005; Yao et al., 2006). In dendrites, stimuli that induce long-term potentiation promote translation of a specific set of mRNAs that include *Arc* (activity-regulated cytoskeleton-associated protein) (Steward et al., 1998), whereas long-term depression-inducing stimuli promote that of different mRNAs such as *IRSp53* (insulin receptor substrate p53) (McEvoy et al., 2007). Whether cue-specific cotranslation of related mRNAs results from the formation of a high-order mRNP granule or simultaneous translation of separate mRNP granules remains to be determined.

### Translation and Human Disease

Although mRNA translation plays a vital role in every cell in the organism, surprisingly, loss-of-function mutations in translation factors and their regulators often result in defects restricted predominantly to the nervous system, for example imbalance in excitatory and inhibitory synapses (Geschwind and Levitt, 2007; Gkogkas et al., 2013). It is plausible that small changes in the amounts of specific proteins in the dendrites and axons have much larger effects than in other tissues. As evidence points increasingly to a key role for local translation in dendrites and axons in neural wiring and maintenance, it is conceivable that dysregulated local translation in these distal neuronal compartments may contribute to, and even underlie, the pathophysiology in these conditions. This concept, however, has not been experimentally validated, as it has not yet been technically feasible to inhibit mRNA translation locally for prolonged time periods in vivo. However, promising progress in this direction is being made. Nonphosphorylatable 4E-BP1 was engineered to be produced by an mRNA with a 3′-UTR of *β-actin* mRNA that targets the mRNA to axons and dendrites (Eom et al., 2003), and a 5′ IRES that permits cap-independent translation (Hellen and Sarnow, 2001), avoiding translational inhibition by its own gene product. This mutant form of 4E-BP1 was synthesized in neuronal axons and dendrites, thereby locally exerting its inhibitory effect on cap-dependent translation (Hsiao et al., 2014). This approach could provide a means to address the important question whether dysfunctional translation at pre- and postsynaptic compartments contributes to neurodegenerative and neurodevelopmental diseases in vivo.

#### Neurodegenerative Diseases

*mRNP Aggregates and ALS*. Dramatic progress in human genetics has revealed a striking link between RBPs and human neurodegenerative disease. For example, mutations in the RBPs, hnRNPA1 and A2B1 (Kim et al., 2013), TDP-43 (Sreedharan et al., 2008), and FUS (Kwiatkowski et al., 2009; Vance et al., 2009) are strongly associated with ALS. These RBPs possess prion-like domains with self-assembling properties (as described above), and intriguingly are found in unusually large aggregates in diseased cells. The finding that the self-assembly ability of these proteins is enhanced by mutations found in ALS patients is very exciting (Kim et al., 2013). Under physiological conditions, many mRNP granules containing these RBPs shuttle between the nucleus and the cytoplasm where they disperse, so that the precise location where the pathological RBPs cause damages to the cell is unknown (Ramaswami et al., 2013). It is likely, however, that these pathological aggregates irreversibly trap physiological submicroscopic mRNP granules, preventing them from being transported to appropriate subcellular compartments including distal axons. As there is evidence that sustained local translation in distal axons is key to neuronal survival (Jung et al., 2012; Kaplan et al., 2009), it is plausible that decreased translation of mRNAs trapped in pathological aggregates might damage distal axons and that the accumulation of such damage causes the death of postmitotic neurons. The upper and lower motor neurons, which are among those that have the longest axons and are prone to degeneration in ALS patients, might be particularly sensitive to pathological mRNP aggregates as mRNP transport may impact them more than neurons with shorter axons.

*eIF2α and Prion Diseases*. Decreased global translational activity is observed in a wide range of neurodegenerative diseases, such as Alzheimer’s, Parkinson’s and prion diseases (Hoozemans et al., 2012; Moreno et al., 2012). In prion-infected mice, which are a model of prion disease, the accumulation of misfolded proteins activates PERK, which represses global translational activity by phosphorylating eIF2α (Moreno et al., 2012) (Figure 2A). Remarkably, decreasing eIF2α phosphorylation in neurons almost completely rescues the failure of synaptic transmission and the death of neurons in these mice, indicating that the repression of global translational activity causes the pathology (Moreno et al., 2012). Intriguingly, a sudden decline in the level of synaptic proteins precedes defective synaptic function and neuronal loss during pathogenesis, suggesting that defective maintenance of the synaptic proteome might be an underlying cause of neurodegeneration.

Considering the evidence that local mRNA translation is required for presynaptic maturation (Taylor et al., 2013) and plasticity (Zhang and Poo, 2002), it is likely that sustained local translation in the presynaptic terminal is required for synaptic maintenance in adult mice. In support of this notion is the finding that translational activity is enhanced by extrinsic cues that axons encounter in vivo (such as BDNF, netrins and semaphorins), either by increasing 4E-BP phosphorylation via the activation of mTORC1 (Campbell and Holt, 2001) or by decreasing eIF2α phosphorylation potentially through inhibition of either PERK or GCN2, or activation of eIF2α phosphatases (Nukazuka et al., 2008). It will be important to determine whether the sustained repression in presynaptic mRNA translation, as opposed to global translational, contributes to neuronal loss in neurodegenerative diseases.

#### Neurodevelopmental Diseases

*Fragile X Syndrome: The Loss of a Translational Brake*. FMRP is a negative regulator of translation, which binds to the coding sequences of polysome-bound mRNAs and stalls the elongating ribosomes (Darnell et al., 2011). Mutations in the human FMRP-coding gene (*Fmr1*) are associated with neurodevelopmental disorders, such as fragile X mental retardation, a disease characterized by intellectual disability, disruptive and autistic-like behavior, epileptic seizures and language deficits (Darnell and Klann, 2013), and autism (Zukin et al., 2009). In the developing mouse brain, FMRP-containing granules localize to dendrites and axons (Christie et al., 2009), and the loss of FMRP function leads to defective formation of pre- and postsynaptic terminals (Darnell and Klann, 2013). These studies suggest that excessive translation due to the loss of a translational brake (FMRP) results in the development of aberrant neuronal connections and the behavioral manifestations of fragile X syndrome in adulthood.

*mTORC1 Hyperactivation and Neurodevelopmental Disorders*. Mutations in other negative regulators of translation have also been implicated in neurodevelopmental disorders. Autism spectrum disorders (ASDs) encompass a wide spectrum of neurodevelopmental diseases characterized by impaired cognition and communication (Geschwind and Levitt, 2007). ASDs are associated with mutations that lead to hyperactivation of the mTORC1 pathway (Kelleher and Bear, 2008). In tuberous sclerosis, which results from mutations in the mTORC1-negative regulators *Tsc1* or *Tsc2*, 85% of affected patients display cognitive impairment tightly linked to autistic features, epilepsy and abnormal or absent speech (Curatolo et al., 2008). Similarly, mutations in the mTORC1-negative regulator *Pten* lead to PTEN hamartoma syndrome, which manifests with neurobehavioral features resembling autism, macrocephaly and language impairment (Zhou and Parada, 2012). Down syndrome, the most common chromosomal abnormality (trisomy of chromosome 21) leading to mental retardation, is also associated with hyperactive mTORC1, and in a mouse model of Down syndrome, hippocampal dendritic translation is increased and can be restored to the basal level by rapamycin treatment (Troca-Marín et al., 2012).

In summary, it has become clear that aberrant fluctuations, both decreases and increases, in translational activity lead to pathologies—the former generally causing neurodegenerative diseases and the latter causing neurodevelopmental diseases, underscoring the notion that balanced mRNA translation is required for normal brain development and function. Despite this association, it is not yet certain whether the etiology of these syndromes lies in the alteration in the global translational activity or local translation at the synapse. The key to understanding the pathophysiology of these syndromes will require the elucidation of the exact location of defective translation, the mechanisms of aberrant translation, and the target mRNAs whose translation is altered.

### Perspectives and Future Directions

#### Local Translation at Single-Molecule Resolution

Genome-wide subcellular transcriptome studies revealed that hundreds and perhaps thousands of different mRNAs localize to axonal growth cones (Deglincerti and Jaffrey, 2012) and dendrites (dendritic spines, where activity-dependent translation occurs) (Cajigas et al., 2012). Although these studies analyzed mRNAs isolated from a population of neurons, not from a single neuron, these results do suggest that many mRNA molecules might coexist in the same axons and dendrites. How can so many mRNAs be accommodated in these small subcellular compartments? Although previous studies have suggested that multiple mRNAs may be stored in the same mRNP granules (Gao et al., 2008), recently developed single-RNA-molecule imaging techniques revealed, unexpectedly, that even mRNAs containing the same class of *cis*-element do not colocalize, but travel individually (Amrute-Nayak and Bullock, 2012; Batish et al., 2012; Mikl et al., 2011). Quantitative analysis showed that each granule contained only one or two mRNA molecules (Mikl et al., 2011). Intriguingly, the mRNA content of individual granules could be regulated, up or down, by neuronal activity and the amount of RBPs (Staufen in this case). This regulation was message-specific (*Map2* but not *β-actin* mRNA was regulated) (Mikl et al., 2011). An extensive analysis of single *β-actin* mRNA molecules in neuronal dendrites showed that proteinaceous components of the mRNP granule mask individual mRNAs and ribosomes making them inaccessible to translational machinery, and that neuronal activity-dependent “unmasking” may be the molecular basis of localized translation (Buxbaum et al., 2014).

These new findings suggest that there is a mechanism to keep mRNP granules physically separate from one another, possibly by inhibiting their intrinsic self-assembling activity through the prion-like domain, and that these mRNPs could form higher-order granules when required (Erickson and Lykke-Andersen, 2011). Evidence that this might be the case in vivo was recently provided by Singer’s laboratory. Using live tissue from a transgenic mouse in which endogenous *β-actin* mRNAs are bound to multiple copies of green fluorescent proteins (up to 48 copies per mRNA), they showed that single *β-actin* mRNA molecules undergo continuous assembly and disassembly, and that individual mRNA molecules are released from high-order granules in a neuronal activity-dependent manner, perhaps for local translation (Park et al., 2014). Considering that neuronal dendrites contain hundreds, even thousands of different mRNAs (Cajigas et al., 2012), there could be numerous mRNPs within these small neuronal subcellular compartments, which can be regulated individually. Mutations in RBP-coding genes, such as *hnRNPA2* that is associated with neurodegenerative diseases (Kim et al., 2013), might cause mRNP granules to overly self-assemble to a degree that cannot be reversed by normal regulatory mechanisms, causing the cell to lose its ability to control local gene function. New insights into the molecular basis of such diversity and the mechanism of mRNP-specific assembly/disassembly at the single-molecule level awaits the invention of new technologies that will allow us to investigate protein composition of single mRNP granules. Combining such techniques with fluorescent in situ RNA sequencing (FISSEQ) (Lee et al., 2014), which visualizes thousands of different RNA molecules within a cell in situ, is an exciting avenue that will lead to the molecular dissection of individual mRNP granules. These results provide a tantalizing intersection of two major themes from the issue as insight into why Location Matters may ultimately emerge from studies harnessing The Power of One—analysis of single mRNAs and mRNPs.

#### Coordinated Protein Synthesis and Degradation

Thousands of different mRNPs will make neuronal growth cones and dendritic spines heavily crowded, indicating that there must be a mechanism that ensures subcellular proteomic homeostasis is maintained. In line with this idea, molecular machines that degrade specific proteins, including the ubiquitin proteasome system (Campbell and Holt, 2001; Chapman et al., 1994; Ehlers, 2003; Patrick et al., 2003; Speese et al., 2003; Zhao et al., 2003) and autophagy machinery (Yang et al., 2013), operate in neuronal axons and dendrites. A surprising finding was that local translation-dependent responses, such as cue-induced growth cone steering and axon regeneration, also require protein degradation, indicating that protein synthesis and degradation are closely linked in signal transduction (Campbell and Holt, 2001; Verma et al., 2005). How these seemingly opposite processes cooperate to mediate the same response remains a puzzle. One possibility is that a spatially restricted, transient rise in the concentration of a protein is key to translating an extrinsic signal into a precise signal within the cell. The growth cone’s ability to steer toward netrin-1 is blocked either by protein synthesis inhibitors or degradation inhibitors (Campbell and Holt, 2001). Newly made β-actin molecules in the side near the gradient source might then have to be degraded before they diffuse throughout the small space within the growth cone, thus confining a rise in the β-actin concentration to the site of actin assembly where it is needed. Alternatively, protein degradation might keep proteins at the minimum level so that a small rise in protein concentration by local translation could generate a meaningful signal. As it was proposed that cue-induced synthesis of proteins with short half-lives, which never reach a steady-state level, is essential in keeping cells responsive to that stimulus (Schwanhäusser et al., 2011), it would be intriguing to see whether proteins newly synthesized in the neuronal subcellular compartments are specifically targeted for degradation after their cue-induced synthesis.

#### Linking Extrinsic Cues to mRNA Specificity

How extrinsic cues are linked to the translation of a specific set of mRNAs is an important question. At least one study directly addressed this question. Flanagan and colleagues showed that deleted in colorectal cancer-1 (DCC1), a single transmembrane cell-surface receptor for netrin-1, directly binds to ribosomes and translation initiation factors, and translation is promoted by ligand binding (Tcherkezian et al., 2010). As ribosomes and initiation factors are components of mRNPs such as stress granules (Erickson and Lykke-Andersen, 2011), their interaction with cell-surface receptors, which can be regulated by ligand-receptor interaction, provides a conceptually appealing mechanism of cue-mRNA specificity. mRNPs containing mRNAs encoding functionally related proteins could be predocked to a specific signal-receiving component, such as cell-surface receptors or adaptor proteins, to allow the swift translation of subcellularly targeted mRNAs in the vicinity of signal reception by bypassing the rate-limiting initiation step.

Another intriguing possibility is intracellular ribosome heterogeneity. It has become clear that the ribosome shows variable compositions and mRNA specificity in organisms ranging from bacteria (Byrgazov et al., 2013) to mammals (Kondrashov et al., 2011). The ribosome filter hypothesis (Mauro and Edelman, 2002) states that the composition of the ribosome determines mRNA-selective translation, and continues to be supported by new pieces of evidence, including ribosomal protein L38 tuning the ribosome toward the *Hox* mRNAs (Kondrashov et al., 2011) and ribosomal proteins S6 and S7 tuning the ribosome toward intron-containing pre-mRNAs (Apcher et al., 2013). It is a tantalizing idea that ribosomes bound to a specific set of mRNAs could be targeted to different subcellular compartments waiting for the right moment to start making specific proteins.

#### Horizontal RNA Transfer

A challenge in regulating gene expression by localized translation in a compartment distant from the nucleus is the long-distance mRNAs must travel. For example, injured axons may need to quickly synthesize a protein, which is not normally needed and thus whose mRNA is not locally stored. Conceptually, this could be overcome if mRNAs could be transferred from a juxtaposed cell. It may be less efficient to transport mRNAs from the cell body to the tip of the axon than to receive them from neighboring cells, such as postsynaptic neurons or perisynaptic glia, and indeed this mechanism has been proposed (Alvarez, 2001). What was a rather provocative hypothesis initially has gained a solid basis because feeding *C. elegans* bacteria harboring small interfering RNAs (siRNAs), 20–24-nucleotide dsRNAs that posttranscriptionally silence gene expression in a sequence-specific manner (Fire et al., 1998), leads to gene silencing in the entire animal, indicating the presence of a mechanism for cell-to-cell RNA transfer (Timmons and Fire, 1998). Furthermore, animals and plants can accept small RNAs from other organisms both in the same and different species and even from the environment (Baulcombe, 2013; Sarkies and Miska, 2013).

Indeed, recent evidence suggests that a similar mechanism might be used to regulate local mRNA repertoires, as the polysome, multiple ribosomes translating a single mRNA, from Schwann cells can be transferred to the segment of an axon that they insulate, a process promoted by axonal injury (Court et al., 2008; Court et al., 2011; Sotelo et al., 2014). Therefore, it is possible that remote subcellular compartments such as neuronal axons may dynamically regulate the composition of locally stored mRNAs by horizontal transfer of mRNP granules depending on extrinsic signals, and this intriguing mechanism may provide an unmatchable agility and flexibility in dynamically regulating local gene function. As it has been shown that cells, notably those in the immune system, use exosomes, secreted vesicles of endocytic origin, to transfer mRNAs to other cells, where they can be translated (Valadi et al., 2007), it will be intriguing to find out whether exosome-mediated horizontal RNA transfer is used to regulate the local transcriptome in neurons.

#### Human Diseases

Advances in sequencing technologies led to the discovery of new biomarkers and risk genes associated with human disease. The main focus of human genetic studies is to link mutations in protein-coding regions, which cause alterations in protein structure and expression, to diseases. Considering that localized translation plays a key role in regulating gene expression and function, mutations in the UTRs that may affect RNA localization and translation should be another focus that merits extensive investigation. For this kind of analysis, it will be essential to systematically categorize and identify mRNA motifs and sequences important for subcellular targeting and translational control. Bioinformatic analysis of subcellularly targeted and/or translating mRNAs will provide a basis for identifying key *cis*-elements and subsequently for designing new therapies to manipulate local translation of specific mRNAs. This avenue may yield novel therapeutic strategies for neurodevelopmental and neurodegenerative diseases (as described above).

Given the strong evidence linking mRNP granules and neurodegenerative diseases, unraveling molecular mechanisms that regulate mRNP granule assembly, disassembly, and clearance will shed fresh light into their pathophysiology. Of equal importance is the distinction between pathological and normal mRNP granules. A deep understanding of mRNP granules may point to a new direction in treating neurodegenerative diseases, by preventing the formation of pathological mRNP aggregates or facilitating their clearance.

Measuring mRNA abundance in diseased cells is another main focus of biomarker discovery. Considering that the abundance of a protein has a stronger correlation with the translational rate of its mRNA than with the mRNA abundance, it will be critical to analyze translating mRNAs (translatome) rather than total mRNAs (transcriptome). Recent genome-wide translational profiling techniques such as ribosome profiling (Ingolia et al., 2009) and immunoprecipitation of activated ribosomes and associated mRNAs (Knight et al., 2012) provide opportunities to reveal mRNAs whose dysregulated translation is associated with disease status. Furthermore, a strong implication of local translation in neurodevelopmental and neurodegenerative diseases indicates that a deeper look into the local translatome in the axon and dendrite may uncover novel mRNAs whose defective local translation caused the pathology. Applying translational profiling strategies to target axons and dendrites in vivo will be a technical challenge, but overcoming this hurdle may shed new light into the links between localized translation and human diseases.

### Conclusions

Although our appreciation of localized translation is most advanced in neurons, these examples may just represent the tip of the iceberg with a resounding message that Location Matters for control of protein expression. Given the close ties with neurodevelopment and neurological disease, insights into how Location Matters will ultimately impact research striving to Decode the Brain.
